# The role of 25-hydroxycholesterol in the pathophysiology of brain vessel dysfunction associated with infection and cholesterol dysregulation

**DOI:** 10.1242/dmm.052145

**Published:** 2025-05-23

**Authors:** Victor S. Tapia, Sarah E. Withers, Ran Zhou, Abigail Bennington, Christopher Hoyle, Frances Hedley, Adam El Khouja, Nadim Luka, Marco Massimo, Siobhan Crilly, Katherine R. Long, Catherine B. Lawrence, Paul R. Kasher

**Affiliations:** ^1^ Division of Neuroscience, School of Biological Sciences, Faculty of Biology, Medicine and Health, Manchester Academic Health Science Centre, The University of Manchester, Manchester M13 9PL, UK; ^2^ Geoffrey Jefferson Brain Research Centre, The Manchester Academic Health Science Centre, Northern Care Alliance National Health Service (NHS) Foundation Trust, The University of Manchester, Manchester M13 9PL, UK; ^3^ Lydia Becker Institute of Immunology and Inflammation, Faculty of Biology, Medicine and Health, Manchester Academic Health Science Centre, The University of Manchester, Manchester M13 9PL, UK; ^4^ Immunology and Inflammation Theme, Translational and Clinical Research Institute, Newcastle University, Newcastle NE2 4HH, UK; ^5^ Division of Cardiovascular Sciences, School of Medical Sciences, Faculty of Medicine, Biology and Health, The University of Manchester, Manchester M13 9PL, UK; ^6^ Centre for Developmental Neurobiology, Institute of Psychiatry, Psychology and Neuroscience, King's College London, London SE1 1UL, UK; ^7^ MRC Centre for Neurodevelopmental Disorders, King's College London, London SE1 1UL, UK

**Keywords:** Cholesterol, 25-hydroxycholesterol, Zebrafish, Intracerebral haemorrhage, Brain endothelium, SARS-CoV-2

## Abstract

The antiviral enzyme cholesterol 25-hydroxylase (CH25H) and its metabolite 25-hydroxycholesterol (25HC), which modulates cholesterol metabolism during infection, have been associated with vascular pathology. Viral infections have been linked to intracerebral haemorrhage (ICH) risk, but the molecular mechanisms leading to ICH via antiviral responses remain unknown. We hypothesised that the CH25H/25HC pathway impacts neuroendothelial integrity in the context of infection-associated ICH. Using a severe acute respiratory syndrome coronavirus 2 (SARS-CoV-2) spike protein-induced zebrafish ICH model and foetal human SARS-CoV-2-associated cortical tissue containing microbleeds, we identified upregulation of CH25H in infection-associated cerebral haemorrhage. Using zebrafish models and human brain endothelial cells, we asked whether 25HC promotes neurovascular dysfunction by modulating cholesterol metabolism. We found that 25HC and pharmacological inhibition of cholesterol synthesis had an additive effect to exacerbate brain bleeding in zebrafish and *in vitro* neuroendothelial dysfunction. 25HC-induced dysfunction was also rescued by cholesterol supplementation *in vitro*. These results demonstrate that 25HC can dysregulate brain endothelial function by remodelling cholesterol metabolism. We propose that CH25H/25HC plays an important role in the pathophysiology of brain vessel dysfunction associated with infection and cholesterol dysregulation in the context of ICH.

## Teaser

**Summary:** The antiviral metabolite 25-hydroxycholesterol dysregulates brain endothelial function by remodelling cholesterol metabolism, thereby providing a mechanistic link between viral infection and brain endothelial dysfunction in conditions such as intracerebral haemorrhage.

## INTRODUCTION

Intracerebral haemorrhage (ICH) is a type of stroke caused by the rupture of brain vessels and subsequent bleeding within the brain parenchyma. Although several ICH risk factors have been described (An et al., 2017), the rare cases in which infection leads to brain vessel rupture are not well understood (Tartarin et al., 2024). Viral infections, such as severe acute respiratory syndrome coronavirus 2 (SARS-CoV-2), herpes zoster, herpes simplex, hepatitis C and dengue, have been associated with the incidence of ICH (Chang et al., 2021; Hauer et al., 2019; Schink et al., 2016; Tseng et al., 2015; Xu et al., 2022). ICH may result from local damage linked to viral encephalitis and brain vasculitis in some of these infections (Chang et al., 2021; Hauer et al., 2019; Nagel and Gilden, 2014). However, reports that flu-like symptoms precede ICH events (Van Etten et al., 2021) suggest that systemic antiviral responses might also be involved. Insights into how antiviral responses, both systemic and local, lead to brain vessel dysfunction will improve the understanding of haemorrhagic stroke pathophysiology.

Alongside adult-onset ICH, foetal brain haemorrhages have also been associated with SARS-CoV-2 infection (Massimo et al., 2023) and antiviral maternal immune activation (Rasile et al., 2022). The effects of antiviral signalling in the developing brain can also be observed in monogenic conditions such as type I interferonopathies. Early post-natal brain haemorrhages have been described for Aicardi-Goutières syndrome 5 (AGS5) (Ramesh et al., 2010; Xin et al., 2011) caused by recessive variants of the viral restriction factor *SAMHD1*. We have previously modelled the cerebrovascular phenotype of AGS5 in zebrafish larvae (Kasher et al., 2015; Withers et al., 2023), reporting a novel link between type I interferon (IFN) signalling, cholesterol dysregulation and susceptibility to brain haemorrhages (Withers et al., 2023). Although others have previously highlighted a link between IFN signalling and inhibition of cholesterol metabolism at both physiological and cellular levels (Robertson and Ghazal, 2016), this has not been previously associated with cerebrovascular defects.

A mechanistic link between antiviral signalling and remodelling of cholesterol metabolism has been reported for the IFN-stimulated enzyme cholesterol 25-hydroxylase (CH25H) and its metabolite 25-hydroxycholesterol (25HC) (Blanc et al., 2013). The antiviral mechanisms of 25HC have been well described, involving the inhibition of cholesterol synthesis and depletion of plasma membrane cholesterol (Blanc et al., 2013; Heisler et al., 2023; Wang et al., 2020). Besides a role in immune defence, recent studies have shown that the CH25H/25HC pathway has a detrimental role in vascular function in certain pathologies, such as atherosclerosis, lung inflammation and experimental autoimmune encephalomyelitis (EAE) (Canfrán-Duque et al., 2023; Madenspacher et al., 2023; Ruiz et al., 2023). Whether CH25H/25HC has a role in cerebrovascular dysfunction associated with ICH remains unknown.

In this study, we hypothesised that cholesterol remodelling induced by 25HC could induce cerebrovascular dysfunction. First, we characterised upregulation of CH25H in SARS-CoV-2 spike protein (spike)-injected zebrafish larvae and human SARS-CoV-2-associated developmental brain haemorrhages. Then, we evaluated the effects of 25HC on zebrafish ICH models and in the human brain endothelial cell (EC) line hCMEC/D3. We found an additive effect between 25HC and pharmacological inhibition of the cholesterol biosynthesis enzyme 3-hydroxy-3-methylglutaryl-coA reductase (HMGCR) by statins, which increased the severity of brain bleeding in zebrafish larvae and brain endothelial dysfunction *in vitro*. 25HC effects were also dependent on cholesterol availability, as cholesterol supplementation rescued the 25HC-induced dysfunction in hCMEC/D3 cells. We propose that the CH25H/25HC pathway could be a relevant factor in infection-triggered brain EC dysfunction.

## RESULTS

### CH25H is expressed in infection-associated developmental intracerebral haemorrhage

**Fig. 1.**
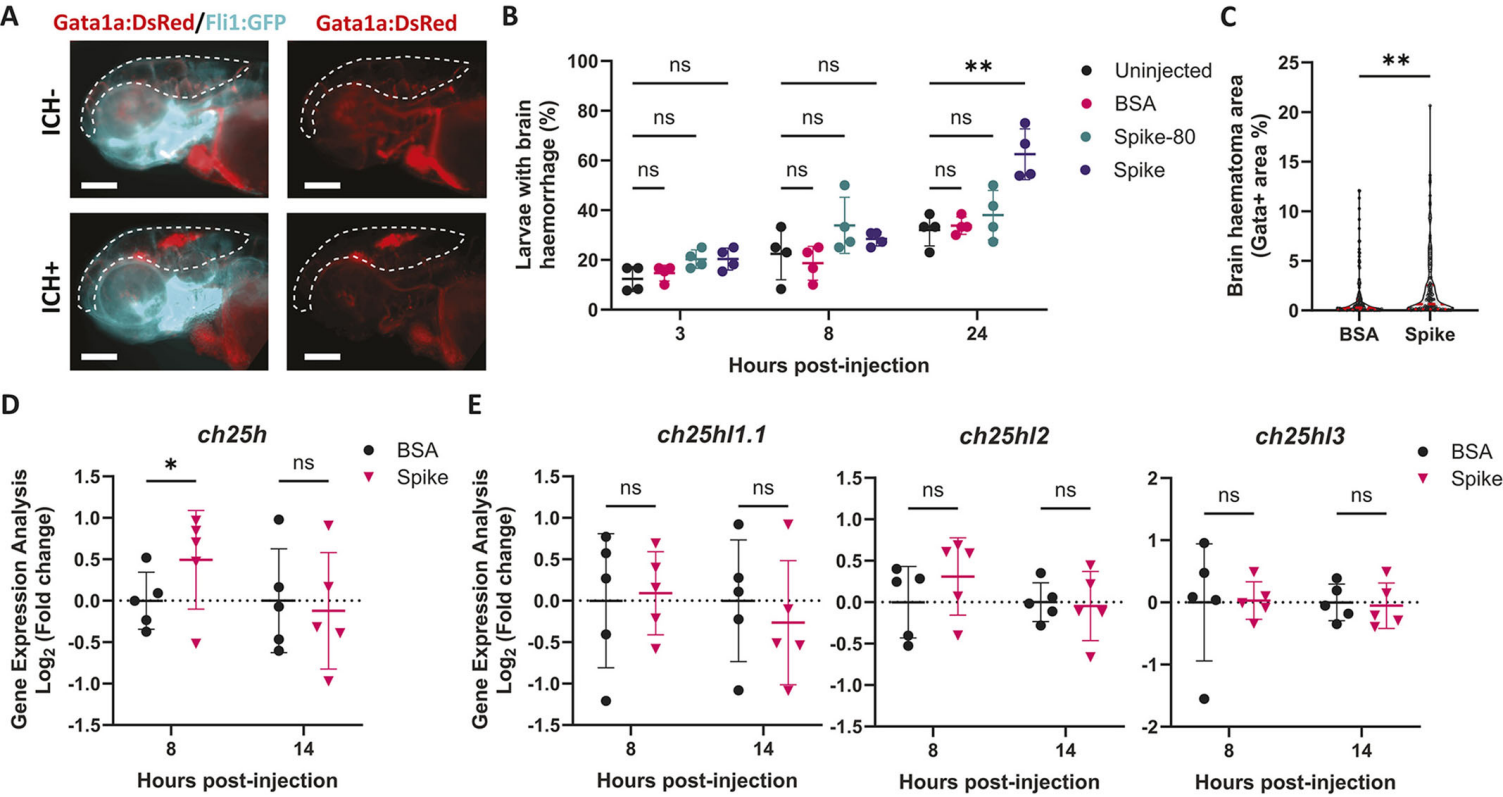
**SARS-CoV-2 spike protein induces *ch25h* upregulation prior to spontaneous brain bleeding in zebrafish larvae.** (A) Representative images of control [intracerebral haemorrhage (ICH)^−^] and haemorrhagic (ICH^+^) *Tg(fli1:EGFP)/Tg(gata1a:DsRed)* 3 days post-fertilisation (dpf) larvae, 24 h after SARS-CoV-2 spike protein (spike) injection into the hindbrain (0.25 mg ml^−1^, 2 nl). Red indicates erythrocytes (*gata1a*^+^) and cyan indicates endothelial cells (*fli1*^+^). Dashed lines indicate the brain area. Scale bars: 250 µm. (B) Time course of ICH^+^ frequency in *Tg(fli:EGFP)/Tg(gata1a:DsRed)* larvae that were uninjected, injected with bovine serum albumin (BSA) control, injected with pre-heated spike at 80^o^C for 30 min (spike-80) or injected with spike conditions (*n*=4, 11-14 embryos per experiment). (C) Haematoma size (Gata^+^ area in brain region) in larvae 24 h post-injection with BSA or spike. Individual embryos are indicated as dots (*n*=146-147, three independent experiments). (D,E) Gene expression was analysed in larval heads 8 and 14 h after BSA or spike injections, for *ch25h* (D), *ch25hl1.1*, *ch25hl2* and *ch25hl3* (E) (30 larval heads pooled per replicate). Data are mean±s.d. (B,C,E) or median±interquartile range (IQR) (C). ns, non-significant; *P<0.05; **P<0.01; determined by repeated measures ANOVA with Dunnett's post-hoc test compared to uninjected (B), Mann–Whitney test (C) or randomised block two-way ANOVA with Sidak's post hoc analysis compared to BSA (D,E).

To ultimately assess the role of the CH25H/25HC pathway in cerebrovascular dysfunction, we wanted to evaluate the expression of CH25H in infection-associated ICH. For this, we took advantage of a SARS-CoV-2 inflammatory model in zebrafish larvae. Although SARS-CoV-2 is not able to replicate in zebrafish (Laghi et al., 2022), injection of the SARS-CoV-2 spike into the hindbrain of zebrafish larvae recapitulates the systemic hyperinflammation observed in coronavirus disease (COVID-19), additionally inducing local brain inflammation and brain haemorrhages (Tyrkalska et al., 2022, 2023). To confirm these findings, we used a zebrafish double transgenic reporter for ECs and erythrocytes [*Tg(fli1:EGFP)/Tg(gata1a:DsRed)*] to evaluate spike-induced brain haemorrhages *in vivo* (Fig. 1A). Spike injections were compared to non-injected, or bovine serum albumin (BSA) or denatured (80°C, 30 min) spike protein (spike-80)-injected control groups. Spike injection did not change brain haemorrhage frequency at 3 or 8 h post-injection but induced a significant increase in frequency at 24 h (Fig. 1B). Furthermore, a significant increase in brain haematoma area was also observed at 24 h post-injection in spike-injected larvae compared to BSA controls (Fig. 1C). We then evaluated the expression of the five *CH25H* homolog genes described in zebrafish (Pereiro et al., 2017). *ch25h* was transiently upregulated by spike injection before the increase in brain haemorrhage frequency, as observed by a significant upregulation at 8 h post-injection and return to basal levels at 14 h (Fig. 1D). In contrast, spike injection had no effect on the other zebrafish homolog genes – *ch25hl1.1*, *ch25hl2* and *ch25hl3* (Fig. 1E) – and *ch25hl1.2* was not detected (Table S1). These findings corroborate previous research indicating that zebrafish *ch25h* is upregulated in response to viral challenge, whereas the other *CH25H* homologs do not exhibit the same antiviral upregulation (Pereiro et al., 2017).

**Fig. 2.**
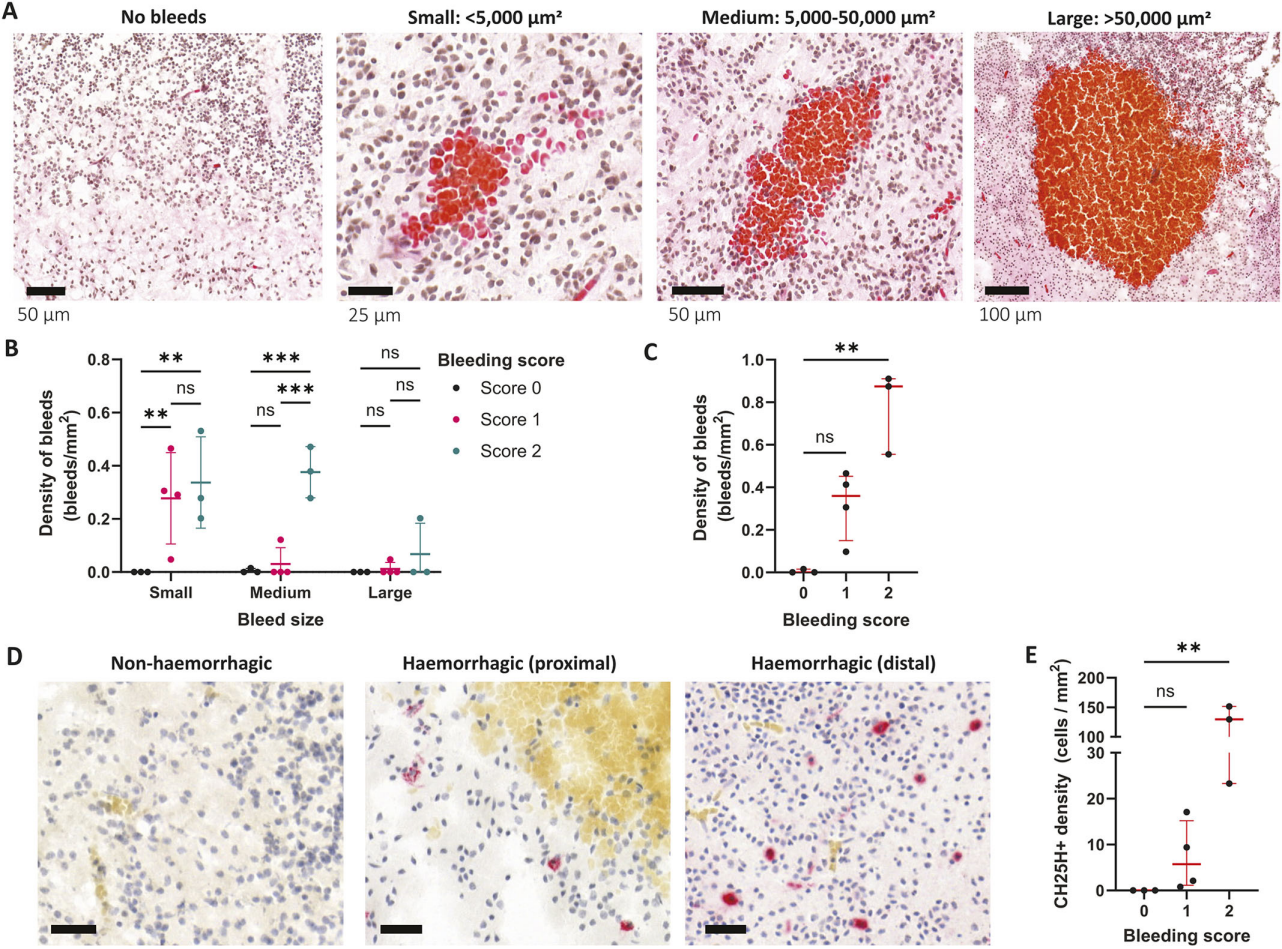
**CH25H expression is associated with human foetal SARS-CoV-2-associated brain microbleeds.** (A) Representative images of microbleeds found in human foetal cortex by Haematoxylin and Eosin staining. Bleeds were classified into small, medium and large sizes according to area. Scale bar size is shown in each image. (B,C) Samples were categorised according to a bleeding score, based on bleed size and total bleed density. Control samples were classified as score 0, and haemorrhagic samples were classified as scores 1 and 2. Density of small, medium and large bleeds (B), and total bleed density (C) is shown for samples with bleeding scores of 0 to 2. (D) Representative images of CH25H staining (pink) and bleeds (yellow) from non-haemorrhagic or haemorrhagic samples, proximal and distal to bleeds. Scale bars: 30 µm. (E) CH25H^+^ cell density was quantified in samples with bleeding scores of 0 to 2. Data are mean±s.e.m. ns, non-significant; **P<0.01; ***P<0.001; determined by two-way ANOVA with Tukey's post hoc analysis multiple comparisons between scores (B), or one-way ANOVA with Kruskal–Wallis post hoc test compared to score 0 (C,E).

To further validate our findings in this zebrafish model, we evaluated CH25H expression in post-mortem human foetal brain samples with SARS-CoV-2-associated brain haemorrhages (Massimo et al., 2023). First, we quantified microbleed number and size in the brain cortical samples, observing a variable size of microbleed, which we classified into three area size categories (Fig. 2A). Measuring the number and size of microbleeds, we classified the cortical samples by a bleeding score, whereby non-haemorrhagic samples had a score of 0, and haemorrhagic samples were divided into scores 1 and 2. Score 2 samples were defined as having significantly more medium-sized bleeds than score 1 samples (Fig. 2B), and significantly more total density bleed than control samples (Fig. 2C). We then evaluated CH25H levels by immunohistochemistry, observing a lack of CH25H^+^ cells in non-haemorrhagic samples and variable numbers of CH25H^+^ cells in haemorrhagic samples, both proximal and distal to haematomas (Fig. 2D). Although CH25H^+^ cells were detected in all haemorrhagic samples, a significant increase in CH25H^+^ cells was observed in samples with a bleeding score of 2 (Fig. 2E).

Next, we explored whether a CH25H antiviral response could be directly observed in cells that form part of the neurovascular unit. We evaluated an endothelial response using the human brain microvasculature cell line hCMEC/D3 and a glial response using primary murine mixed glia culture. We detected significant upregulation of *CH25H* in hCMEC/D3 cells exposed to poly(I:C), a synthetic double-stranded RNA analogue that mimics viral infections, and to IFNβ (Fig. S1A,B). Similarly, we also detected *Ch25h* upregulation in a murine mixed glia culture, which consist of approximately 80% astrocytes, 10% microglia and 10% oligodendrocyte progenitor cells (Pinteaux et al., 2002), exposed to poly(I:C) and IFNβ (Fig. S1C). Overall, our data indicate that CH25H upregulation is part of an antiviral response in SARS-CoV-2-associated haemorrhages in both developing zebrafish and human brains, and that CH25H upregulation can be directly induced by antiviral signalling in neuroendothelial and glial cells.

### 25HC increases the severity of bleeding in a statin-dependent zebrafish ICH model

We next aimed to evaluate whether the CH25H metabolite 25HC could exacerbate cerebrovascular dysfunction. Inhibition of Hmgcr using statins induces spontaneous brain haemorrhages in zebrafish larvae (Eisa-Beygi et al., 2013; Li et al., 2017; Withers et al., 2023). This is caused by defects in neuroendothelial prenylation-dependent signalling, which depends on intermediate metabolites of the cholesterol synthesis pathway (Eisa-Beygi et al., 2013; Zhang and Casey, 1996). We have previously shown that reduced expression of *hmgcrb* in a zebrafish model of type I interferonopathy is associated with increased susceptibility to statin-induced ICH (Withers et al., 2023). As 25HC decreases HMGCR levels by transcriptional inhibition, inhibiting sterol regulatory element-binding protein2 (SREBP2; also known as SREBF2) (Saito et al., 2023) and inducing HMGCR proteolysis (Faulkner et al., 2024), we hypothesised that 25HC would also exacerbate an ICH phenotype.

**Fig. 3.**
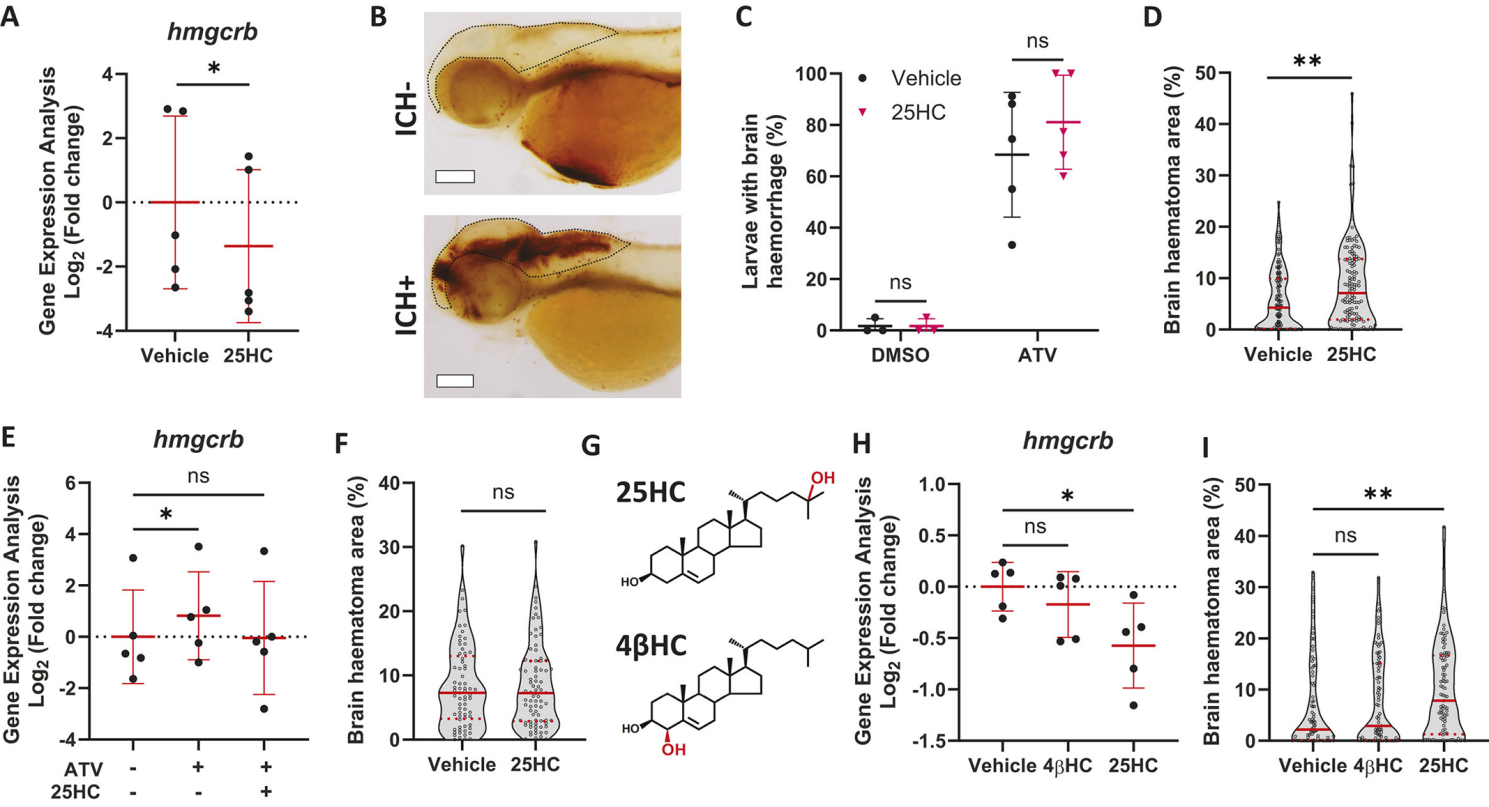
**25HC worsens brain haemorrhage expansion in a statin-induced ICH zebrafish model.** (A) *hmgcrb* expression in 2 days post-fertilisation (dpf) wild-type (WT) zebrafish larvae incubated with 25-hydroxycholesterol (25HC; 25 μM, 24 h) (15 embryos pooled per replicate). (B-D) WT larvae were incubated in Atorvastatin (ATV; 1 µM) at 28 h post-fertilisation (hpf) and intravenously injected with 25HC (1 nl, 5 µM) at 32-36 hpf. The next day, larvae were stained with o-Dianisidine. Representative images of larvae without (ICH^−^) and with (ICH^+^) brain haemorrhage are shown (B). Dotted lines indicate the brain area. Scale bars: 150 µm. ICH^+^ frequency per experiment (C) and brain haematoma area per larvae (D) were quantified. Individual embryos are indicated as dots (*n*=128 embryos, five independent experiments). (E) *hmgcrb* expression in 2 dpf WT larvae incubated with 25HC (25 µM) and ATV (1 µM) for 24 h (15 embryos pooled per replicate). (F) *bbh* zebrafish larvae were injected with 25HC (1 nl, 5 µM) at 32-36 hpf. After 24 h, larvae were stained with o-Dianisidine and brain haematoma area was quantified. Individual embryos are indicated as dots (*n*=69-82 embryos, three independent experiments). (G) Comparison of 25HC and 4β-hydroxycholesterol (4βHC) structures. (H) Expression of *hmgcrb* in 2 dpf WT larvae incubated with 4βHC or 25HC (25 µM, 24 h) (15 embryos pooled per replicate). (I) WT larvae were incubated in ATV (1 µM) at 28 hpf and injected with 4βHC or 25HC (1 nl, 2.5 µM) at 32-36 hpf. The next day, larvae were stained with o-Dianisidine and haematoma area was quantified. Individual embryos are indicated as dots (*n*=87-93 embryos, three independent experiments). Data expressed as mean±s.d. (A,C,E,H) or median±IQR (D,F,I). ns, nonsignificant; *P<0.05; **P<0.01; determined by paired two-tailed *t*-test (A), randomised block two-way ANOVA with Sidak's post-hoc test compared to control (C), Mann–Whitney test (D,F), randomised block one-way ANOVA with Dunnett's post-hoc test compared to control (E,H), or Kruskal–Wallis test with Dunn's post-hoc test compared to control (I).

Wild-type (WT) zebrafish larvae that were incubated with 25HC (25 µM, 24 h) exhibited significantly reduced transcription of the *HMGCR* zebrafish homolog gene *hmgcrb* (Fig. 3A), confirming that 25HC has a similar transcriptional effect in zebrafish. To evaluate the effects of 25HC and Atorvastatin (ATV) on brain bleeding in zebrafish, ATV-induced haemorrhages were identified by o-Dianisidine staining (Fig. 3B) (Withers et al., 2023). Following ATV incubation, 25HC was injected (1 nl, 1 mM) directly into the bloodstream through the duct of Cuvier (Benard et al., 2012). 25HC injections alone did not increase ICH occurrence in untreated larvae; however, in ATV-incubated larvae, we observed a non-significant increase in ICH frequency (Fig. 3C). To assess the extent of cerebral bleeding, we next evaluated brain haematoma area in the same animals, showing that, in ATV-treated embryos, 25HC induced a significant increase in bleed area (Fig. 3D). This zebrafish ICH model demonstrates that 25HC exacerbates ATV-induced neurovascular instability.

We then evaluated whether the ATV and 25HC additive effect was dependent on HMGCR inhibition. ATV incubation increased the expression of *hmgcrb*, a feedback response induced through the activation of SREBP2 (Göbel et al., 2019), and 25HC co-incubation inhibited this process (Fig. 3E). We confirmed the disruption of this feedback response by evaluating HMGCR protein levels in human brain ECs using western blotting. HMGCR protein levels increased in hCMEC/D3 cells treated with ATV (1 µM, 16 h) but decreased in those with 25HC exposure (5 µM, 16 h) (Fig. S2A,B). This suggested that 25HC increased the sensitivity to ATV by decreasing HMGCR levels. To confirm whether the 25HC phenotype was dependent on ATV inhibition of HMGCR, we repeated the experiment in an alternative ICH zebrafish model. The homozygous *bbh* zebrafish mutant expresses a hypomorphic mutation in the *βpix* (also known as *arhgef7b*) gene, which leads to dysfunctional neuroendothelium, resulting in a comparable ICH phenotype to that of the ATV model independently of Hmgcr activity (Liu et al., 2007). 25HC injections in *bbh* larvae caused no differences in haematoma size (Fig. 3F). This demonstrated that 25HC effects on neurovascular stability are seemingly dependent on Hmgcr inhibition. To confirm this, we compared 25HC to another oxysterol, 4β-hydroxycholesterol (4βHC), which shares a similar structure (Fig. 3G) but has no effect on SREBP2 activity (Heisler et al., 2023). This was corroborated by *hmgcrb* expression analysis in WT zebrafish larvae, which was significantly inhibited by 25HC incubation but not by 4βHC (Fig. 3H), and by HMGCR protein analysis in hCMEC/D3 cells, which decreased in a higher magnitude upon 25HC exposure than in response to 4βHC (Fig. S2C,D). When both oxysterols were injected alongside ATV incubation, only 25HC increased haematoma area in WT zebrafish larvae (Fig. 3I). These results revealed that 25HC increases the risk of ATV-induced ICH by decreasing *hmgcrb* gene expression and HMGCR protein levels.

### 25HC remodels cholesterol metabolism in human brain ECs

**Fig. 4.**
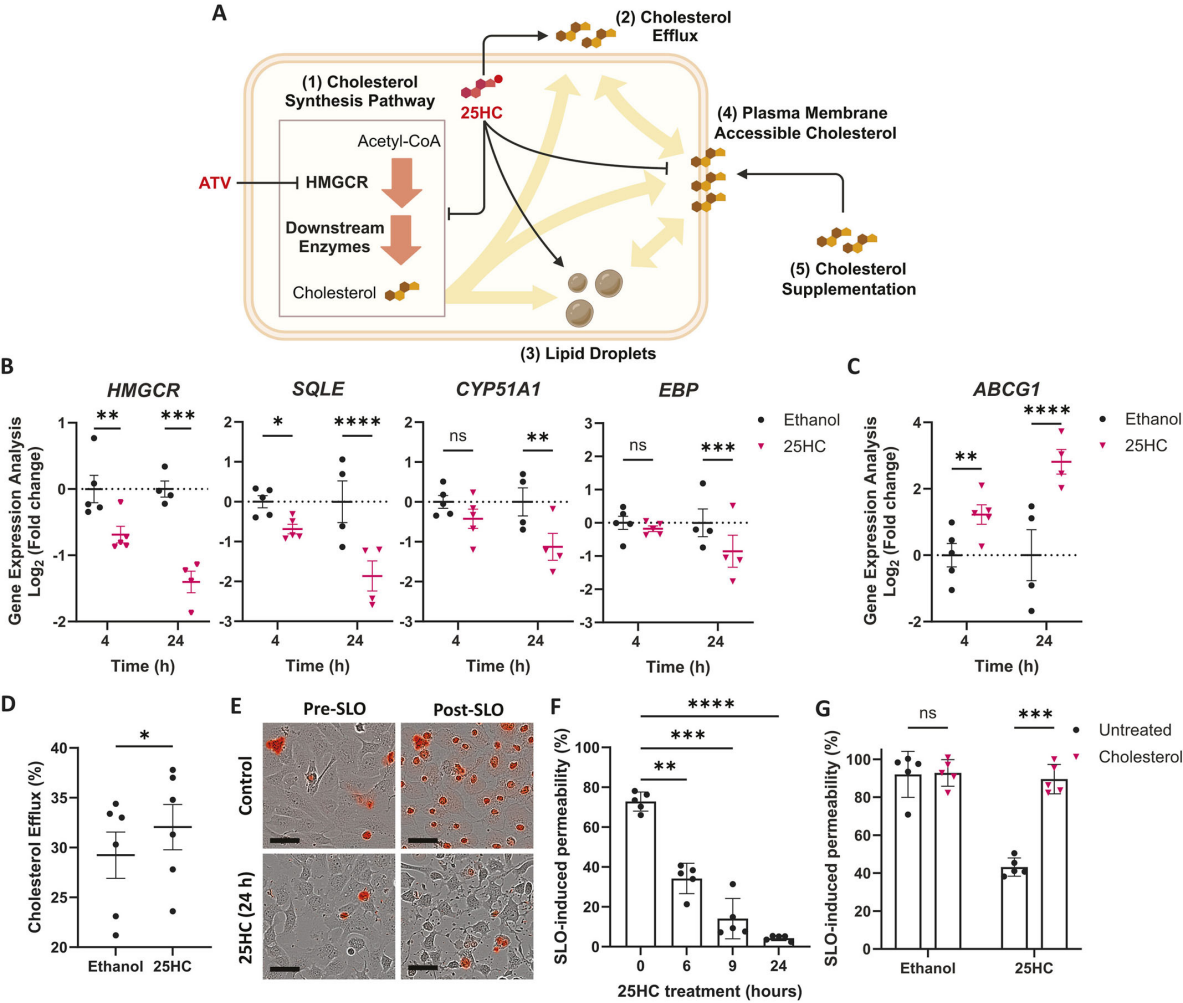
**25HC remodels cholesterol metabolism in human brain endothelial cells.** (A) Cellular cholesterol remodelling induced by 25HC. 25HC inhibits cholesterol synthesis (1), promotes cholesterol efflux (2), and induces the internalisation and storage of cholesterol in the form of lipid droplets (3). These changes lead to the depletion of plasma membrane-accessible cholesterol (4), which can be rescued with cholesterol supplementation (5). (B,C) Expression of *HMGCR*, *SQLE*, *CYP51A1*, *EBP* (B) and *ABCG1* (C) genes in hCMEC/D3 cells after 25HC treatment (5 μM, 4 and 24 h). (D) hCMEC/D3 cells were loaded with fluorescent cholesterol (1 h) before 25HC treatment (5 μM, 16 h). Cholesterol efflux in fresh medium (for 4 h) was measured by fluorescence. (E,F) hCMEC/D3 cells were pre-treated with 25HC (5 μM, 0 to 24 h) before incubation with streptolysin O (SLO). Membrane permeability was measured by To-Pro-3^+^ uptake (red signal) before and after SLO; representative images (E) and quantification (F) are shown. Scale bars: 37.5 µm. (G) Permeability of hCMEC/D3 cells, pre-treated with 25HC (5 μM, 6 h) and then with soluble cholesterol (80 μM, 1 h), after SLO incubation. Data expressed as mean±s.d. ns, nonsignificant; *P<0.05; **P<0.01; ***P<0.001; ****P<0.0001; determined by paired two-tailed *t*-test (D), randomised block one-way ANOVA with Dunnett's post hoc test compared to 0 µM (F), or randomised block two-way ANOVA with Sidak's post-hoc test compared to control (B,C,G).

In addition to decreasing HMGCR levels, 25HC modulates cholesterol metabolism through several other mechanisms (Fig. 4A), including the downregulation of other cholesterol synthesis enzymes (Nishimura et al., 2005), stimulation of cholesterol efflux (Cashikar et al., 2023), and internalisation and storage of cholesterol in the form of lipid droplets (Abrams et al., 2020; Wang et al., 2020), which altogether lead to the depletion of plasma membrane-accessible cholesterol (Heisler et al., 2023; Wang et al., 2020). To assess whether 25HC also influenced these mechanisms in the context of neurovascular stability, we employed an *in vitro* approach using the hCMEC/D3 cell line (Fig. 4A). First, we evaluated the expression of several genes encoding cholesterol synthesis enzymes, including *HMGCR*, in hCMEC/D3 cells at 4 and 24 h after 25HC exposure (Fig. 4B). The expression of *HMGCR* and *SQLE* was significantly decreased after both 4 and 24 h, while the expression of *CYP51A1* and *EBP* was significantly decreased after 24 h (Fig. 4B). These results, alongside the observed decrease in HMGCR protein levels (Fig. S2A,B), suggest that 25HC supresses multiple points of the cholesterol synthesis pathway in brain ECs.

25HC also regulates cholesterol efflux through activation of LXR transcription factors (Cashikar et al., 2023; Saito et al., 2023). ABCG1 is an LXR target involved in cholesterol efflux (Kennedy et al., 2005). Using the same hCMEC/D3 samples (Fig. 4B), we detected significant upregulation of *ABCG1* expression after exposure to 25HC for both 4 and 24 h (Fig. 4C). We then assessed whether this upregulation translated into functional changes in cholesterol efflux, by incubating hCMEC/D3 cells with fluorescent cholesterol and measuring its efflux. 25HC pre-treatment (16 h) induced a modest, but significant, increase in cholesterol efflux (Fig. 4D).

In addition to its role in transcriptional regulation, 25HC directly promotes cholesterol internalisation, which leads to its storage in lipid droplets (Abrams et al., 2020; Wang et al., 2020). To assess this, we stained for lipid droplets using the fluorescent dye BODIPY (Qiu and Simon, 2016). Surprisingly, no lipid droplets were detected in either control or 25HC-treated hCMEC/D3 cells (Fig. S3A). To confirm that our assay was sufficiently sensitive, we demonstrated that lipid droplets could be detected in hCMEC/D3 cells overloaded with cholesterol (80 µM, 16 h) (Fig. S3A) and in primary murine macrophages (Fig. S3B), a cell type known to accumulate lipid droplets (Rosas-Ballina et al., 2020). These results suggest that 25HC does not promote lipid droplet formation in brain ECs despite its known role in cholesterol internalisation (Abrams et al., 2020; Wang et al., 2020).

We next asked whether these changes in synthesis and efflux influenced cellular cholesterol levels. One important antimicrobial mechanism induced by 25HC is the depletion of plasma membrane-accessible cholesterol levels (Heisler et al., 2023; Wang et al., 2020; Zhou et al., 2020). Using streptolysin O (SLO), a microbial toxin that depends on accessible cholesterol to form membrane pores (Zhou et al., 2020), we indirectly evaluated accessible cholesterol levels by measuring SLO-induced permeability through uptake of the small dye To-Pro-3 (Fig. 4E). SLO permeabilised the membrane of hCMEC/D3 cells, and this permeability was inhibited by 25HC pre-treatment in a time-dependent manner (Fig. 4F). This suggested that 25HC quickly induced the depletion of accessible cholesterol. To confirm this, cells were supplemented with soluble cholesterol (80 µM, 1 h) after 25HC treatment. Cholesterol supplementation rescued the permeability phenotype (Fig. 4G), thereby confirming that the reduction in permeability was due to a decrease in accessible cholesterol levels. Altogether, these results suggest that 25HC remodels cholesterol metabolism in brain ECs by suppressing cholesterol synthesis and enhancing cholesterol efflux, but not via lipid droplet formation, which is associated with reduced accessible cholesterol levels.

### 25HC-induced dysfunction of human brain ECs depends on cholesterol metabolism

**Fig. 5.**
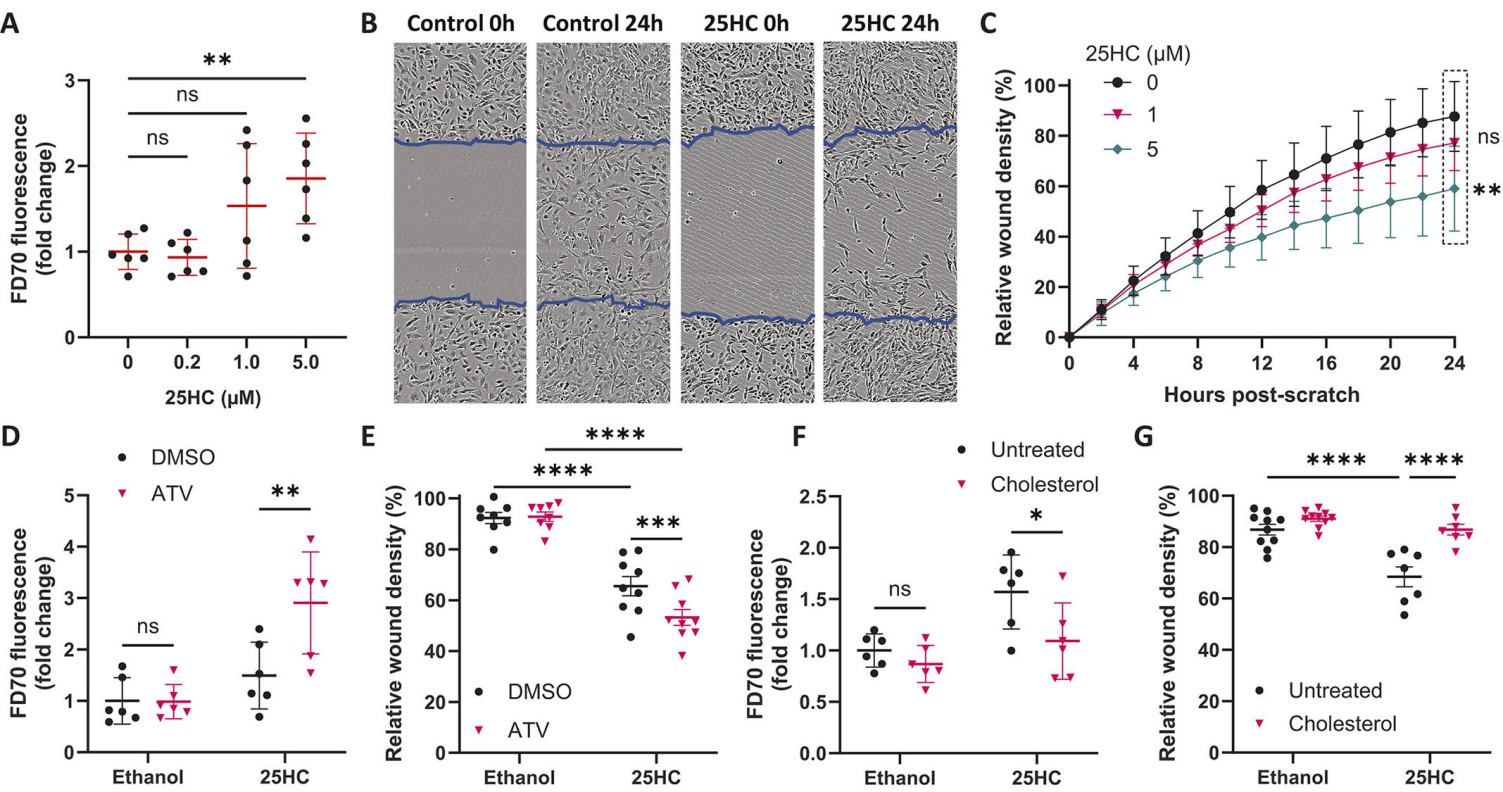
**25HC dysregulates the function of human brain endothelial cells.** (A) Permeability of fluorescein-conjugated dextran 70 kDa (FD70) in hCMEC/D3 cell monolayer pre-treated with different concentrations of 25HC (0-5 μM, 24 h). (B,C) hCMEC/D3 cell migration in a scratch assay; cells were pre-treated with different concentrations of 25HC (0-5 μM, 24 h before scratch). Representative images are shown for cells 0 and 24 h after scratch (B; 5 μM 25HC). Blue lines show the initial scratched area. Scale bars: 100 µM. Migration was quantified as relative wound density (C; *n*=10). (D) FD70 permeability was analysed in hCMEC/D3 cells treated with 25HC (5 μM, 24 h) and ATV (1 μM, 24 h). (E) Cell migration at 24 h post-scratch of hCMEC/D3 cells pre-treated with 25HC (5 μM, 24 h) before scratch and treated with ATV (1 µM, 24 h) after scratch. (F) FD70 permeability was analysed in hCMEC/D3 cells pre-treated with 25HC (5 μM, 14 h) and then supplemented with soluble cholesterol (80 µM, 1 h). (G) Cell migration at 24 h post-scratch of hCMEC/D3 cells pre-treated with 25HC (5 μM, 24 h) and then supplemented with cholesterol (80 µM, 2 h) before scratch. Data expressed as mean±s.d. ns, nonsignificant; *P<0.05; **P<0.01; ***P<0.001; ****P<0.0001; determined by randomised block one-way ANOVA with Dunnett's post-hoc test compared to 0 µM (A), matched measures two-way ANOVA with Dunnett's post-hoc test compared to 0 µM (C), or randomised block two-way ANOVA with Sidak's post-hoc test compared to control (D-G).

As 25HC exacerbated the neurovascular instability phenotype in zebrafish (Fig. 3), we next questioned whether 25HC would also affect the barrier function of brain ECs *in vitro*. For this, we analysed the permeability of fluorescein dextran 70 kDa (FD70) through a hCMEC/D3 monolayer, a method previously used to assess hCMEC/D3 barrier function (Afonso et al., 2008). 25HC induced a concentration-dependent decrease in barrier function, with 25HC (5 µM) treatment leading to a significant 85% increase in FD70 permeability (Fig. 5A). Although barrier function relies on the formation of cell–cell junctions, the ability to maintain and remodel these junctions is also important for angiogenic endothelial migration (Bentley et al., 2014). To further investigate the impact of 25HC on endothelial function, we used a previously established scratch assay to evaluate hCMEC/D3 cell migration (Thurgur et al., 2022) (Fig. 5B). One day pre-treatment with 25HC decreased cell migration in a concentration-dependent manner (Fig. 5C). These effects were neither associated with cell death (Fig. S4A-C) nor with a decrease in cell density, as 25HC treatment did not affect total cell numbers (Fig. S4D) and inhibition of cell migration in the scratch assay persisted even with cell cycle arrest induced by mitomycin C (Fig. S4E).

We next aimed to determine whether HMGCR inhibition was involved in the 25HC-induced dysfunction of hCMEC/D3 cells. As in the zebrafish ICH model, HMGCR inhibition by statins has been shown to decrease *in vitro* EC barrier function at micromolar concentrations (Aslam et al., 2019; Li et al., 2017). Therefore, we hypothesised that 25HC and ATV would also have an additive effect in hCMEC/D3 cells. ATV treatment alone (1 µM, 24 h) did not affect barrier function, whereas ATV and 25HC co-treatment significantly increased permeability in comparison to 25HC alone (Fig. 5D). A similar effect between 25HC and ATV was observed in the scratch assay. ATV alone had no effect on cell migration, but it significantly exacerbated inhibition of cell migration in 25HC pre-treated cells 24 h post-scratch (Fig. 5E). These results demonstrate that, similar to our zebrafish model, 25HC-induced hCMEC/D3 dysfunction is dependent on HMGCR inhibition.

As our *in vitro* analysis suggested that 25HC also decreases cholesterol synthesis and cholesterol accessible levels in brain ECs, we also aimed to determine whether the effects of 25HC on endothelial function are dependent on cellular cholesterol. For this, hCMEC/D3 cells were supplemented with soluble cholesterol (as in Fig. 4G) after being pre-treated with 25HC. Using the FD70 permeability assay, we observed that cholesterol supplementation rescued the effect of 25HC on barrier function (Fig. 5F). Similarly, cholesterol supplementation also rescued 25HC inhibition of cell migration. hCMEC/D3 cells were pre-treated with 25HC for 1 day and then supplemented with cholesterol before the scratch. This treatment significantly increased the migration of 25HC-treated cells (Fig. 5G). Altogether, these results suggest that the 25HC-induced dysfunction of brain ECs is also mediated by a decrease in cholesterol availability.

## DISCUSSION

Here, we propose that the CH25H/25HC pathway represents an important component of brain EC dysfunction associated with infection. Our findings in zebrafish larvae and human foetal samples suggest that CH25H is upregulated in viral-associated developmental brain haemorrhages. In these samples, most human foetal haemorrhages occurred between 12 and 14 weeks post-conception (Massimo et al., 2023), a critical window for neurovascular development marked by endothelial junction protein upregulation (Andrews et al., 2018; Virgintino et al., 2004). Similarly, in our zebrafish ICH models, bleeding occurs between 2 and 3 days post-fertilisation (dpf) (Eisa-Beygi et al., 2013; Liu et al., 2007; Tyrkalska et al., 2022), coinciding with brain angiogenesis and barrier function development (Quinonez-Silvero et al., 2020). These results suggest that an antiviral response, including upregulation of CH25H, affects the critical steps of brain vascular development.

**Fig. 6.**
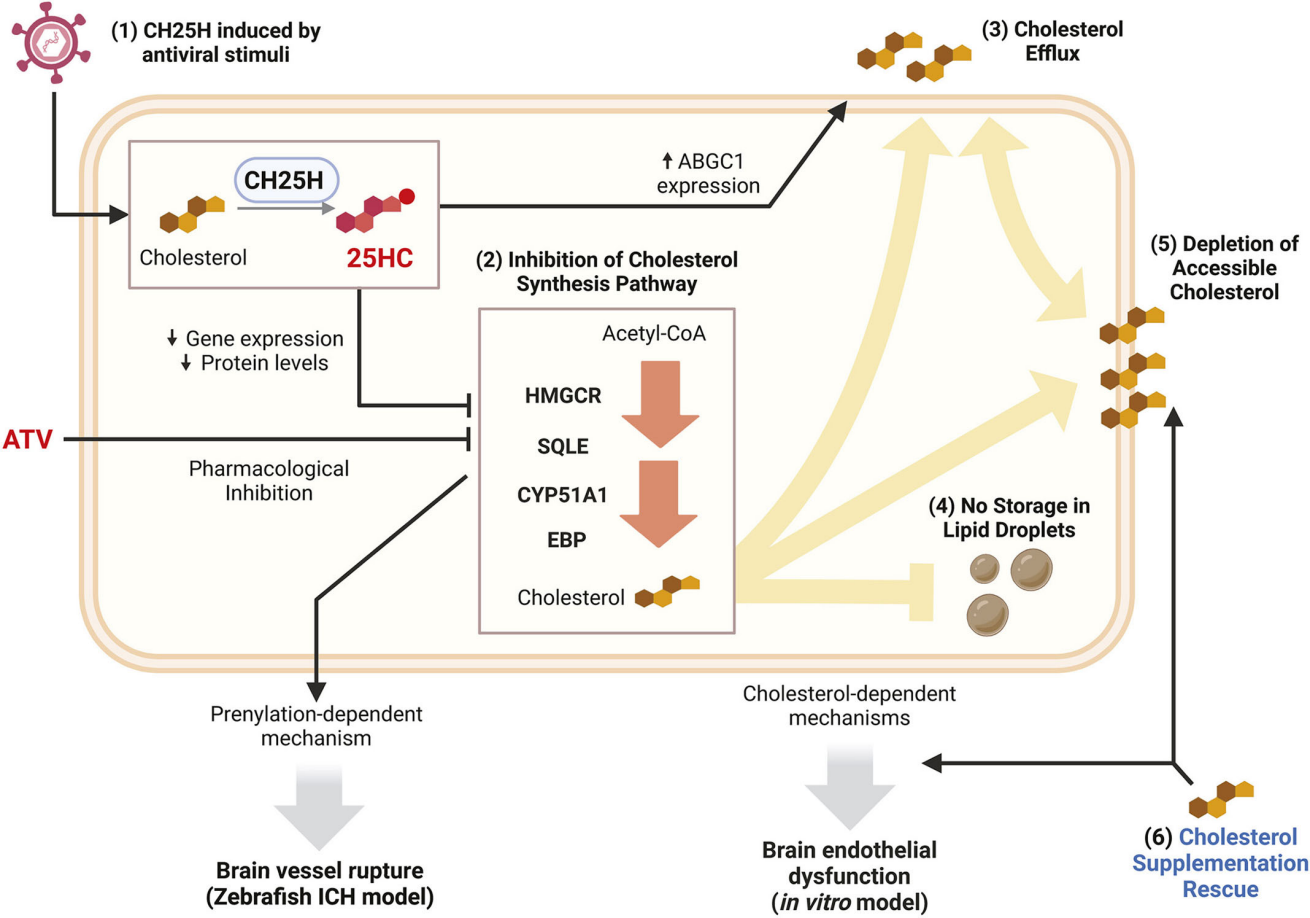
**25HC remodels cholesterol metabolism and function of brain endothelial cells.** (1) CH25H upregulation was detected in SARS-CoV-2-associated ICH in zebrafish and human foetal brain tissue, as well as in hCMEC/D3 cells exposed to antiviral stimuli [poly(I:C) and IFNβ]. (2) 25HC treatment induced the downregulation of cholesterol synthesis enzymes at both mRNA and protein levels. In zebrafish and hCMEC/D3 cells, this downregulation had an additive effect when combined with pharmacological inhibition of HMGCR by ATV, leading to brain vessel rupture (ICH) in zebrafish and decreased barrier function and cell migration in hCMEC/D3 cells. (3) 25HC treatment in hCMEC/D3 cells also increased cholesterol efflux, which was associated with upregulation of ABCG1. (4) Internalisation of cholesterol into lipid droplets was not observed in 25HC-treated hCMEC/D3 cells. (5) The changes in cholesterol synthesis and efflux in hCMEC/D3 cells were associated with the depletion of plasma membrane-accessible cholesterol. (6) Cholesterol supplementation rescued the levels of accessible cholesterol and mitigated the decrease in barrier function and cell migration induced by 25HC in hCMEC/D3 cells.

We show that the CH25H metabolite, 25HC, can induce brain EC dysfunction *in vitro* and exacerbates ICH in a zebrafish model. These findings align with previous research linking the CH25H/25HC pathway to vascular inflammation seen in atherosclerosis, EAE and lung injury (Canfrán-Duque et al., 2023; Madenspacher et al., 2023; Ruiz et al., 2023). Although these previous studies have primarily associated the CH25H/25HC pathway with ER stress (Madenspacher et al., 2023) or regulation of inflammatory pathways (Canfrán-Duque et al., 2023; Ruiz et al., 2023), our data highlight cholesterol remodelling as a key process controlling brain endothelial function. 25HC modulated cholesterol metabolism by regulating cholesterol enzyme levels, promoting cholesterol efflux and reducing accessible cholesterol levels in the plasma membrane. Moreover, 25HC-induced brain EC dysfunction was linked to HMGCR activity and cholesterol availability (Fig. 6).

Our findings are consistent with previous reports highlighting the importance of cholesterol homeostasis for EC function, as dysregulation of cholesterol synthesis or transport has been shown to compromise angiogenesis and barrier function (Koponen et al., 2020; Noghero et al., 2012; Tan et al., 2020; Zhou et al., 2004). Endothelial dysfunction has previously been associated with cholesterol-dependent defects in cell–cell junctions (Baumgartner et al., 2014; Tan et al., 2020) and cholesterol-dependent endothelial signalling (Noghero et al., 2012). 25HC may also disrupt neurovascular responses to blood flow, as cholesterol in the plasma membrane is crucial for endothelial signalling in shear stress responses (Park et al., 1998; Yamamoto et al., 2020). Cholesterol remodelling could also affect other processes dependent on intermediate metabolites, such as protein prenylation, which depends on isoprenoids produced by HMGCR (Zhang and Casey, 1996). The vascular instability induced by ATV in our *in vitro* and zebrafish models has been linked to prenylation defects in endothelial GTPases (Aslam et al., 2019; Eisa-Beygi et al., 2013; Tan et al., 2021). It is possible that the additive effect between ATV and 25HC could also have been mediated by defective prenylation in our models, as decreased prenylation has also been proposed as a potential antiviral mechanism for 25HC (Li et al., 2023).

While in this study we used exogenous 25HC in zebrafish and *in vitro* models to study the effects of cholesterol remodelling on brain endothelial function, future investigations should assess the role of CH25H upregulation and endogenous 25HC production in neurovascular function. In response to inflammation, CH25H is upregulated and 25HC levels increase within the brain, as observed in murine models involving lipopolysaccharide challenge (Waltl et al., 2013) and tauopathy neurodegeneration (Toral-Rios et al., 2024). Endothelial CH25H expression may regulate neurovascular function during antiviral responses, as we observed *CH25H* upregulation in hCMEC/D3 cells treated with poly(I:C) and IFNβ (Fig. S1A,B). Notably, brain endothelial CH25H upregulation has been detected *in vivo* in response to brain inflammation (Chen et al., 2020; Ruiz et al., 2023), and endothelial CH25H has been shown to modulate immune responses during EAE progression (Ruiz et al., 2023). Given the reported paracrine effects of 25HC (Abrams et al., 2020; Blanc et al., 2013; Liu et al., 2013), other cell types may also contribute to 25HC production within the neurovascular unit. We observed an increase in *Ch25h* expression in mixed glia culture in response to antiviral stimuli (Fig. S1C). Interestingly, secretion of 25HC has been detected *in vitro* in both microglial (Cashikar et al., 2023) and astrocyte-conditioned (Zhu et al., 2019) media. Future studies should explore whether glial-derived 25HC influences neurovascular function, similar to effects reported in atherosclerosis whereby macrophage-derived 25HC promotes vascular disease progression (Canfrán-Duque et al., 2023).

Our study used foetal human brain samples and zebrafish larval ICH models; however, the mechanisms involving viral infection as a risk factor for adult ICH may differ from those in the developing brain. Animal models of infection-triggered brain haemorrhages are required to understand this process. Brain haemorrhages in adult mice have been observed following Japanese encephalitis and dengue virus infections (Amaral et al., 2011; Tripathi et al., 2021), although associated with severe encephalitis. Brain microbleeds reported in mice infected with SARS-CoV-2 were also associated with brain infection, vasculitis and disruption of the brain–blood barrier (Qiao et al., 2024). Whether these models resemble ICH cases triggered by milder flu-like infections (Van Etten et al., 2021) is unknown. Mild viral infection may only trigger adult ICH in combination with other risk factors and/or co-morbidities. Interestingly, hypocholesterolaemia is a known ICH risk factor (Lee et al., 2024; Sun et al., 2019; Valappil et al., 2012; Wang et al., 2013), and statins have long been debated as potential contributors to ICH risk (Bétrisey et al., 2024; Collins et al., 2004; Mayerhofer et al., 2022; Sabouret et al., 2022). Because our results suggest that the effects of 25HC in brain ECs are dependent on cholesterol remodelling, it is possible that infection-induced upregulation of the CH25H/25HC pathway could interact with hypocholesterolaemia and statin use in the development of adult-onset ICH.

In conclusion, we have reported the upregulation of CH25H in viral-associated developmental brain haemorrhages in a zebrafish model and in human foetal brain samples. Using *in vitro* and zebrafish models, we have demonstrated that 25HC dysregulates brain EC function by remodelling cellular cholesterol. Our results highlight a novel association between 25HC, cholesterol remodelling and brain EC dysfunction.

## MATERIALS AND METHODS

### Zebrafish husbandry

Zebrafish were raised and maintained at The University of Manchester Biological Services Unit under standard conditions, as previously described (Westerfield, 2000). Adult zebrafish husbandry was approved by The University of Manchester Animal Welfare and Ethical Review Board. All zebrafish experiments were performed according to UK Home Office regulations (PPL: PP1968962). Adults were housed in mixed-sex tanks with a recirculating water supply maintained at 28°C under a 14 h/10 h light/dark cycle, as previously described (Westerfield, 2000). Wild-type AB, double transgenic *Tg(fli1a:GFP)^y1^/(gata1a:DsRed)^sd2^* (Delov et al., 2014; Traver et al., 2003) and βpix mutant *bbh* (*bbh*^m292^) (Liu et al., 2007) adult zebrafish lines were used. Fertilised eggs were collected following natural spawning and incubated at 28°C in fresh E3 medium, except embryos injected with SARS-CoV-2 spike protein, which were kept at 26°C until 48 h post-fertilisation (hpf) to delay development. The embryos were staged according to standard guidelines (Kimmel et al., 1995). Larvae were anaesthetised with 0.02% tricaine methanesulfonate (MS222; Sigma-Aldrich) before injections and imaging. After termination of the experiment, all embryos were killed before protected status (5 dpf) using a lethal dose of MS222 (4%) and freezing at −20°C.

### Zebrafish larval ICH models

For the SARS-CoV-2 spike 1 protein model, *Tg(fli1a:GFP)^y1^/(gata1a:DsRed)^sd2^* larvae were kept at 26°C, dechorionated at 24 hpf and incubated with 0.3% N-phenylthiourea (Sigma-Aldrich) to inhibit melanogenesis. At 48 hpf, larvae were injected in the hindbrain with 2 nl SARS-CoV-2 spike S1 protein (0.25 mg ml^−1^) (SinoBiologicals, 40591-V08H3; molecular mass, 116 kDa) or BSA (0.43 mg ml^−1^) (Sigma-Aldrich; molecular mass, 66.4 kDa) in PBS supplemented with 0.05% Phenol Red. Following hindbrain injections, larvae were kept at 28°C for 24 h and brain haemorrhages were imaged *in vivo*.

For the 25HC and ATV model, *z*ebrafish WT larvae were dechorionated and treated by immersion with ATV 1 µM (Merck) at 28 hpf. Control groups included vehicle hydroxypropyl-β-cyclodextrin (HβCD) in all experiments. ATV-treated WT or *bbh* embryos at 32-36 hpf were injected with 1 nl 25HC or 4βHC (0.5 or 1 mM), 2.25% HβCD, 0.05% Phenol Red in PBS through the duct of Cuvier (Benard et al., 2012). To visualise bleeds, embryos were stained at 54 hpf using an o-Dianisidine (Sigma-Aldrich) protocol, as previously described (Eisa-Beygi et al., 2013). Stained embryos were mounted in 50% glycerol PBS and imaged. For quantitative PCR (qPCR) analysis, embryos were treated by immersion with 25 µM 25HC or 4βHC, alongside 0.11% HβCD, at the same time as ATV.

Imaging was performed in a Leica M165FC light stereo microscope with DFC7000T camera and processed using LASX software (version 3.3.3.16958). Images were analysed using ImageJ, brain area (excluding the eyes) was manually selected, and threshold tool was used identically in all conditions to quantify brain haemorrhage area. Injections and manual selection of brain area were conducted unaware of treatment.

### Human foetal cortical samples

Human foetal tissues were obtained from the Human Development Biology Resource (HDBR), provided by the Joint Medical Research Council/Wellcome Trust (MR/R006237/1). Clinical investigation was conducted according to the principles expressed in the Declaration of Helsisnki, and ethical approval was provided by HDBR REC references 23/NE/0135 and 23/LO/0312. The HDBR provided fresh tissue from foetuses aged 9-21 post-conception weeks. Brains were fixed for at least 24 h at 4°C in 4% paraformaldehyde (PFA) in 120 mM phosphate buffer (pH 7.4). Brain cortexes were then treated with sucrose (15% and 30% sucrose solution sequentially for 24 h each) and embedded in optimal cutting temperature (OCT) compound, before 20 μm-thick sections were cut using a cryostat, as previously described (Massimo et al., 2023).

### Staining of human foetal samples

To visualise microbleeds, slides were stained with a Haematoxylin and Eosin (H&E) standard protocol and mounted with DPX mounting medium. For CH25H immunohistochemistry, antigen retrieval was performed by placing slides in a 97.5°C water bath in Tris-EDTA (pH 9.0) for 20 min. Non-specific binding was blocked with PBS with 5% goat serum, 0.3% Triton X-100 and 0.1% Tween 20 for 1 h. Anti-CH25H antibody (Aviva System Biology, OABF01697) was diluted 1:2000 in blocking solution and incubated on slides overnight at 4°C. Slides then were incubated with biotinylated goat anti-rabbit secondary antibody (Vector Laboratories, BA-1000) diluted in TBST buffer (Tris-buffered saline with 0.1% Tween 20 and 0.1% BSA) at 1:400 for 90 min at room temperature. Slides then were stained with an alkaline phosphatase ABC-AP kit (Vector Laboratories, AK-5000), following the manufacturer’s instructions. Finally, slides were counterstained with Haematoxylin and mounted with DPX. Slides were imaged using a 3D Histech Pannoramic 250 Flash Slide Scanner. Images were analysed using the software QuPath (version 0.5.0). Bleed and total brain areas were manually selected, and CH25H^+^ cells were selected using a positive cell detection command. Haemorrhagic samples were classified as score 1 and 2 based on the size and density of bleed. Score 2 samples had higher density of medium, larger bleeds and higher total density of bleeds than score 1 samples.

### Cell culture

The immortalised human cerebral microvascular EC line hCMEC/D3 cells (Merck, SCC066) were cultured in endothelial cell growth medium MV (Promocell, C-22020) and PenStrep (100 units ml^−1^ penicillin and 100 μg ml^−1^ streptomycin) at 37°C in a humidified atmosphere containing 5% CO_2_. Flask, plates and well inserts were pre-coated with rat tail collagen type I (Merck; 1:100 in PBS) at 37°C for 1 h, before hCMEC/D3 seeding. Cells were passaged at 100% confluency using Trypsin-EDTA solution (Sigma-Aldrich) and were used in experiments until passage 15. hCMEC/D3 cells were seeded at a density of 71,000 cells cm^−2^ overnight in all assays, with the exception of lipid droplets, permeability and scratch assays (specific cell density stated in their subsections).

All animal procedures to obtain primary murine cells were performed according to UK Home Office regulations (PPL: PP4035628) and approved by The University of Manchester Animal Welfare and Ethical Review Board. Murine mixed glia cultures were prepared as previously described (Hoyle et al., 2022). Brains were isolated from 2- to 4-day-old C57BL/6 mouse pups (Envigo). Brain tissue was mechanically digested, and cells were maintained in Dulbecco's modified Eagle medium (DMEM), 10% foetal bovine serum (FBS; Life Technologies) and PenStrep until 80% confluence was reached (12 days). Cultures were then re-seeded at a density of 52,000 cells cm^−2^ and culture for two further days.

Murine primary bone marrow-derived macrophages (BMDMs) were prepared as previously described (Hoyle et al., 2022). Marrow cells were flushed from femurs. Red blood cells were lysed with ACK lysis buffer (Lonza), and BMDMs were generated by culturing the resulting marrow cells in DMEM, 10% FBS and PenStrep, supplemented with 30% L929 mouse fibroblast-conditioned medium for 7 days. Primary BMDMs were seeded overnight at a density of 150,000 cells cm^−2^ before the experiment and cultured in DMEM, 10% FBS and PenStrep.

Cells were treated with high-molecular mass poly(I:C) (InvivoGen, tlrl-pic), human IFNβ (Tonbo Biosciences, 21-8699), murine IFNβ (R&D Systems, 12401-1), 25HC (Sigma-Aldrich, H1015), soluble cholesterol (Sigma-Aldrich, C4951), Staurosporine (Cell Guidance System, SM97), Mitomycin C (Roche, 10107409001) or ATV (Merck, SML3030), at concentrations and times stated in figure legends. Poly(I:C) was transfected into hCMEC/D3 cells with Lipofectamine 3000 (Invitrogen). When used as a carrier, control groups included ethanol, dimethyl sulfoxide or Lipofectamine at the same concentration as for treated groups.

### qPCR

Total RNA was pooled groups of zebrafish larvae (*n*=30 larval heads, *n*=15 full larvae), extracted from hCMEC/D3 cells (12-well plates) or murine mixed glia culture (six-well plates) using a standard TRIzol (Invitrogen) method. Complementary DNA (cDNA) was synthesised from 800 ng RNA as previously described (Withers et al., 2023). qPCR was performed on a StepOne Plus Real Time PCR machine (Applied Biosystems). cDNA samples were analysed using Power SYBR Green Mastermix (Applied Biosystems) and primers (Thermo Fisher Scientific). Gene expression was normalised by geometric averaging of two internal control genes. Reference genes used were *hprt1* and *actb2* for zebrafish samples, and *HPRT1/Hprt1* and *18S* (also known as *RNA18SN/Rn18s*) for human and murine samples, respectively. A Taqman (Applied Biosystems) protocol and probes were used for *hmgcrb* and *sqlea* zebrafish genes, using *hprt1* as a reference gene. A list of primers and Taqman probes is provided in Table S2.

### Western blotting

Western blot analysis was performed on hCMEC/D3 cell lysates using antibodies against HMGCR (Novus Biological, NBP2-61617; 1:1000 dilution), full-length and cleaved caspase-3 (Abcam, ab32351; 1:500 dilution), and β-actin (Sigma-Aldrich, A3854; 1:10,000 dilution). Samples were run on 8% or 12% SDS-polyacrylamide gels. Gels were transferred using a Trans-Blot^®^ TurboTM Transfer System (Bio-Rad) before blocking with 5% BSA in PBST (PBS, 1% Tween 20) for 1 h at room temperature. Membranes were washed and incubated (4°C) overnight in primary antibody in PBST with 0.1% BSA. Following this, membranes were washed and incubated with horseradish peroxidase-conjugated secondary antibodies (Dako) in PBST with 0.1% BSA for 1 h at room temperature. Finally, membranes were washed, incubated in ECL Western Blotting Detection Reagent (GE Life Sciences) and imaged using a G:BOX gel doc system (Syngene). Densitometry was performed using ImageJ.

### Cholesterol efflux assay

hCMEC/D3 cells were seeded in 96-well plates overnight. A Cholesterol Efflux Assay Kit (Abcam, ab196985) was used, following manufacturer instructions, treating cells with 25HC alongside equilibration buffer incubation (16 h). Fluorescence readings were measured using a CLARIOstar Plus plate reader (BMG Labtech).

### Lipid droplet analysis

hCMEC/D3 cells (seeding density, 35,000 cells cm^−2^) or BMDMs were seeded on glass coverslips in 24-well plates. After treatments, cells were fixed in 4% PFA for 15 min and stained with BODIPY 493/503 (Thermo Fisher Scientific), 1 µg ml^−1^ in PBS for 10 min. Nuclei staining was performed with 4′,6-diamidino-2-phenylindole (DAPI), and, after drying, coverslips were mounted with Prolong gold antifade reagent (Thermo Fisher Scientific). Images were collected on a Leica TCS SP8 AOBS upright confocal using a 63× objective. Images were then analysed for background removal with ImageJ.

### SLO assay

hCMEC/D3 cells were seeded in 96-well plates overnight and treated as previously stated. After cell treatments, medium was changed to Opti-MEM reduced serum medium (Thermo Fisher Scientific, 11058021) with 0.5 µM To-Pro-3 (Thermo Fisher Scientific, T3605). SLO (Sigma-Aldrich, SAE0089) was activated with 20 mM dithiothreitol (30 min, 37°C) before adding to cells at 2 U µl^−1^. Images were captured before SLO addition and every 15 min after SLO addition using an IncuCyte ZOOM System (Essen Bioscience) with a 20×/0.61 NA S Plan Fluor objective. Then, 90 min after SLO addition, lysis solution (Promega, G1780) was added to capture an image with 100% permeability. Images were automatically analysed using a Top-Hat segmentation using IncuCyte software (Essenbio), which gave optimal detection for To-Pro-30^+^ cells that ran identically in all conditions.

### Permeability assay

hCMEC/D3 cells were seeded overnight at 50,000 cells per well in CellQart 24-well inserts with 0.4 µm pore size (Sterlitech, 9320412) and treated as previously stated. After cell treatments, medium was changed to Opti-MEM reduced serum medium, adding FD70 (0.1 mg ml^−1^; Sigma-Aldrich, 46945) inside the CellQart insert. Then, 30 min later, fluorescence of medium outside the CellQart insert was measured using a Fluorstar reader (BMG Labtech).

### Scratch assay

hCMEC/D3 cells were seeded in six-well plates overnight and pre-treated with 25HC for 24 h before re-seeding for scratch assay. hCMEC/D3 cells were seeded at 40,000 cells per well in 96-well ImageLock plates (Essen BioScience) and left to adhere for 4 h before scratch. During that time, cells were pre-treated with 25HC, 5 µg ml^−1^ mitomycin or soluble cholesterol for 1 or 2 h before scratch. Scratch wound injury was carried out using a 96-pin IncuCyte WoundMaker Tool (Essen BioScience). Cells were then washed twice with PBS and replaced with medium. Phase contrast images were acquired at 2 h intervals for a period of 24 h using an Incucyte Zoom Live Cell Analysis system with a 4×/3.05 NA Plan Apo OFN25 objective. The 96-well Cell Migration Software Application Module (Essen BioScience) was used to quantify relative wound density, as previously described (Thurgur et al., 2022).

### Statistical analysis and data presentation

Statistical analysis was performed using GraphPad Prism (v10.2). Data were presented as single data points with mean±s.d., except for violin plots with single data points with median±interquartile range (IQR). Experimental replicates (*n*) were defined as experiments performed on embryo clutches produced by different zebrafish adult pairs, individual zebrafish embryos, individual human donors, different hCMEC/D3 passages and individual mouse donors for primary cells. Experimental replicates were matched when measured several times (repeated measures ANOVA) or obtained from the same embryo clutches, hCMEC/D3 passages or mouse donor (randomised block ANOVA or paired two-tailed *t*-test). Data were assessed for normal distribution using Shapiro–Wilk normality test. Parametric data were analysed using paired two-tailed *t*-test or one-way ANOVA with Dunnett's post-hoc test. Non-parametric data were analysed using Mann–Whitney test or Kruskal–Wallis test with Dunn's post-hoc test. Two-factor data were analysed by two-way ANOVA with Sidak's post-hoc test or with Tukey's post-hoc test. Time course data were analysed by two-way ANOVA with Dunnett's post-hoc test.

## Supplementary Material

10.1242/dmm.052145_sup1 Supplementary information
