# Supplementary material for: The role of 25-hydroxycholesterol in the pathophysiology of brain vessel dysfunction associated with infection and cholesterol dysregulation
Source: Dis Model Mech. 2025 May 23;18(9):dmm052145. doi: 10.1242/dmm.052145 (PMC12128615; doi:10.1242/dmm.052145)
Supplement: Supplementary information [file dmm-18-052145-s1.pdf]

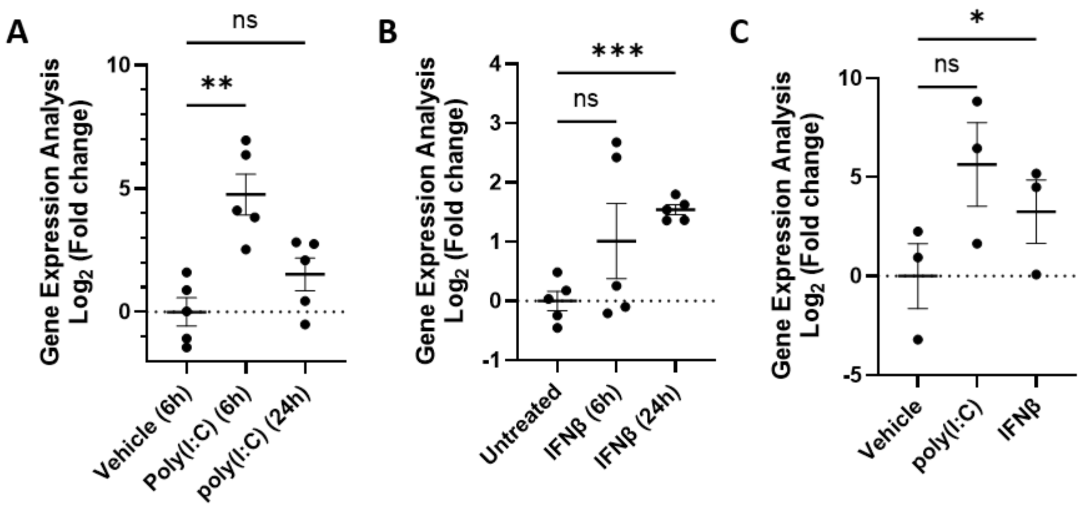

Fig. S1. *CH25H* upregulation in hCMEC/D3 cells and mixed glia culture in response to antiviral stimuli. A-B *CH25H* expression in hCMEC/D3 cells treated with poly(I:C) (30 µg ml^{-1}) (**A**) and human IFNβ (20 ng ml^{-1}) (**B**) for 6 and 24h. **C** *Ch25h* expression in mouse mixed glia culture treated with poly(I:C) (30 µg ml^{-1}) and mouse IFNβ (1 U $µl^{-1}$) for 24 h. Data expressed as mean ± SD. *, $p < 0.05$; **, $p < 0.01$; ***, $p < 0.001$; ns, nonsignificant, determined by randomized block one-way ANOVA with Dunnet's post hoc test compared to control

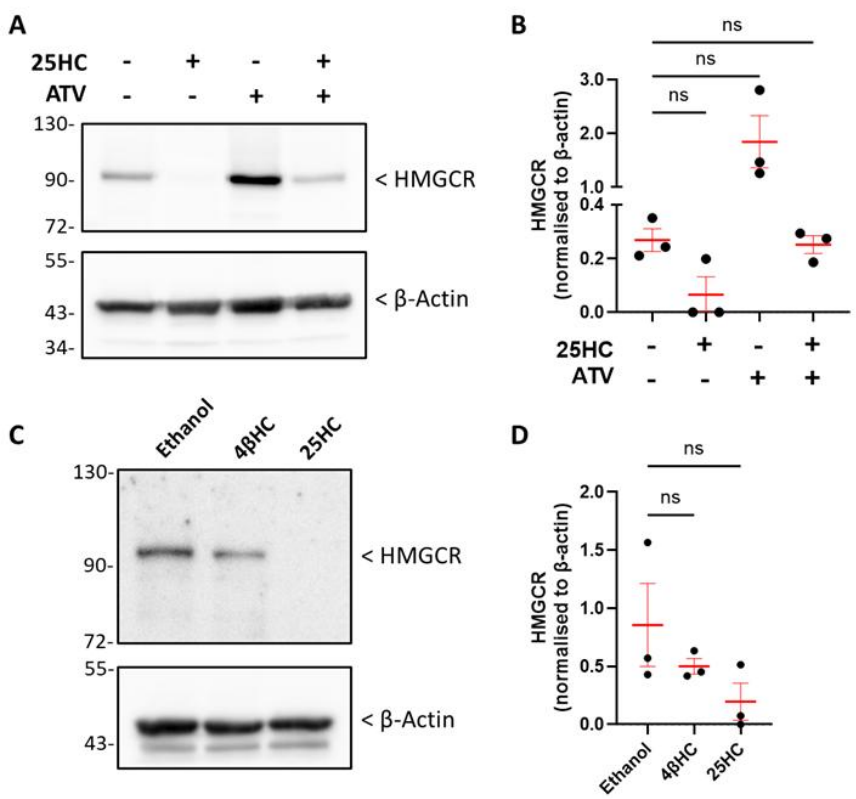

Fig. S2. ATV and 25HC regulate HMGCR protein levels in hCMEC/D3 cells. HMGCR protein levels and β-actin as loading control were analysed by Western Blot in hCMEC/D3 cells after 25HC (5 µM), ATV (1 µM) and 4βHC (5 µM) treatment for 16 h. Representative images (**A**, **C**) and densitometry analysis (**B**, **D**) are shown. Data expressed as mean ± SD. ns, nonsignificant, determined by randomized block one-way ANOVA with Dunnet's post hoc test compared to control.

Disease Models & Mechanisms • Supplementary information

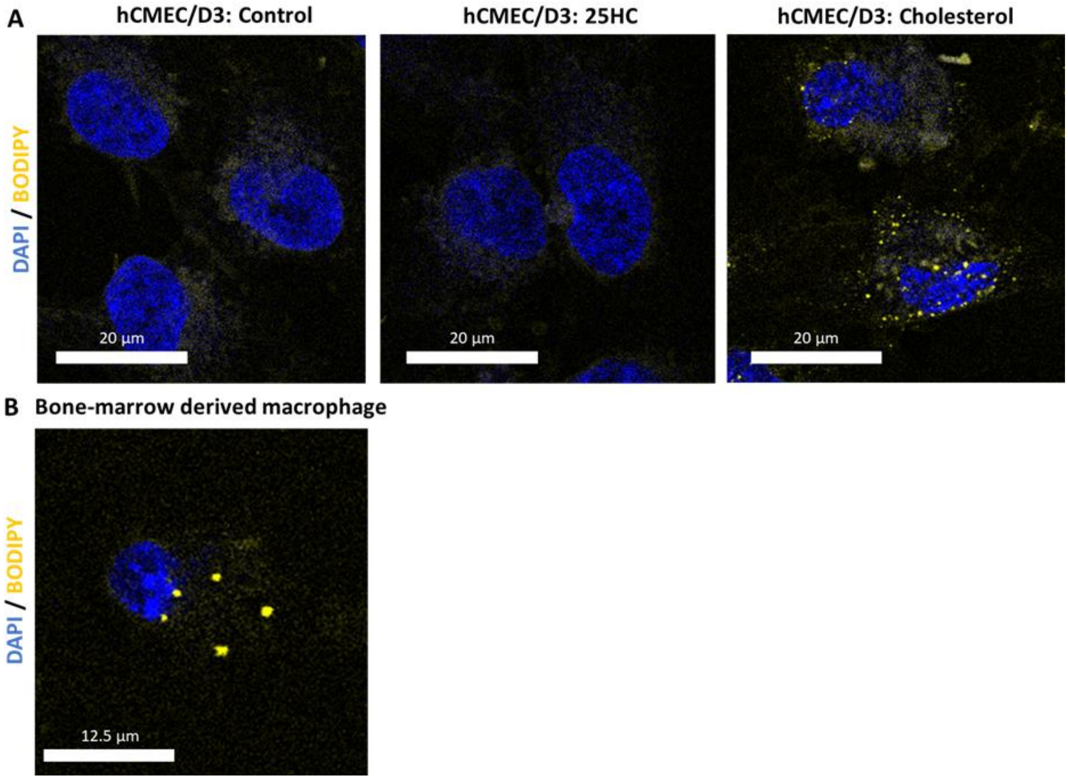

Fig. S3. Lipid droplet analysis in hCMEC/D3 cells and macrophages. A hCMEC/D3 cells treated with 25HC (5 µM, 16h) or soluble cholesterol (80 µM, 16h) were stained with BODIPY (yellow) and DAPI (nuclei, blue) to evaluate lipid droplets. Representative pictures (of two independent replicates) of two or three cells per group are shown. **B** Untreated bone-marrow derived macrophages were stained with BODIPY (yellow) and DAPI (nuclei, blue) to evaluate lipid droplets. Representative picture (of two independent replicates) of one cell is shown.

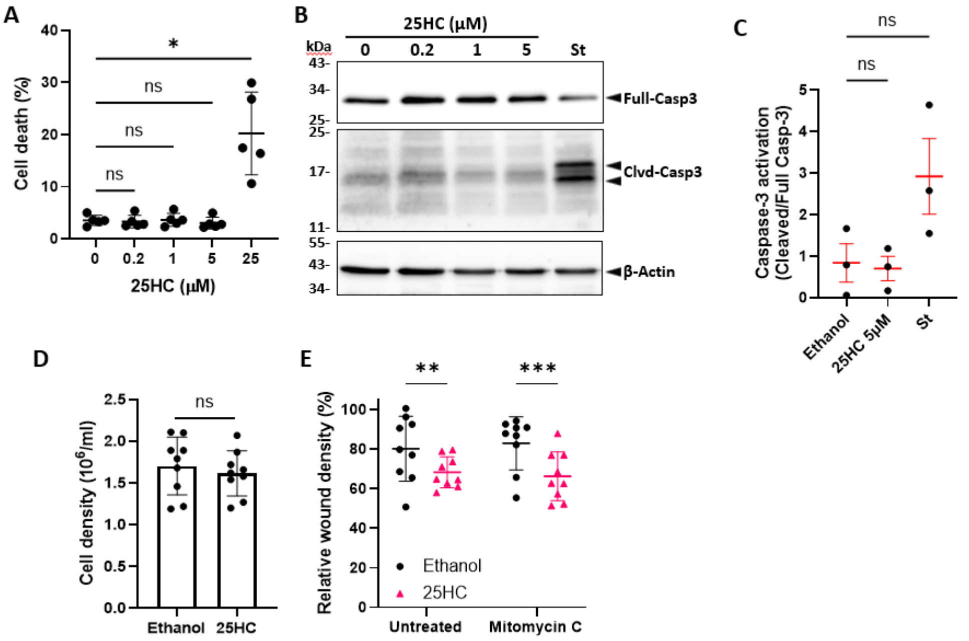

Fig. S4. 25HC (5µM) has no effect on hCMEC/D3 cell death and proliferation. A hCMEC/D3 cells were pre-treated with 25HC (0 - 25 µM) for 24 h, and cell death was assessed by loss of membrane integrity, measuring ToPro3+ uptake. **B-C** Western blot analysis of hCMEC/D3 cell lysates pre-treated with 25HC (0-5 µM, 24 h), compared to staurosporine 5 µM (St) as apoptosis-positive control. Blots for full caspase-3 (FL-Casp3), activated cleaved caspase-3 (Clvd-Casp3) and β-actin as loading control. Representative image (**B**) and densitometry analysis (**C**) are shown. **D** hCMEC/D3 cell density after treatment with 25HC (5 µM, 24 h), counted using a haematocytometer after trypsin detachment. **E** hCMEC/D3 cell migration 24 hours post-scratch, pre-treated with 25HC (5 µM, 24 h before scratch) and then mitomycin D (5 µg/ml, 2 h before scratch). Data expressed as mean ± SD. *, $p < 0.05$; **, $p < 0.01$; ***, $p < 0.001$; ns, nonsignificant, determined by randomized block one-way ANOVA with Dunnet's post-hoc test compared to 0 µM (**A**, **C**), determined by paired t-test (**D**), or randomized block two-way ANOVA with Sidak's post-hoc test compared to control (**E**).

Disease Models & Mechanisms • Supplementary information

Table S1. Threshold cycles (Ct) and melting temperatures (Tm) of housekeeping and ch25h homologs genes in 2 days post-fertilisation zebrafish.

Well	Target Name	C_T	Tm1	Tm2	Tm3
A1	*hprt1*	23.43	82.17		
A2	*hprt1*	23.20	82.17		
A3	*hprt1*	23.91	82.02		
B1	*actb2*	20.95	76.06		
B2	*actb2*	20.96	75.91		
B3	*actb2*	20.78	75.91		
C7	*ch25h*	29.90	81.58		
C8	*ch25h*	29.96	80.54		
C9	*ch25h*	29.89	80.93		
D7	*ch25hl1.1*	25.64	81.28		
D8	*ch25hl1.1*	25.90	81.13		
D9	*ch25hl1.1*	25.79	81.54		
E7	*ch25hl1.2*	Undetermined	64.74	84.41	81.73
E8	*ch25hl1.2*	Undetermined	65.93	83.82	73.23
E9	*ch25hl1.2*	Undetermined	70.10		
F7	*ch25hl2*	30.59	84.11		
F8	*ch25hl2*	30.74	83.67		
F9	*ch25hl2*	30.43	83.36		
G7	*ch25hl3*	28.26	79.79		
G8	*ch25hl3*	28.55	77.85		
G9	*ch25hl3*	28.31	77.70		

Table S2. qPCR primers and probes

Zebrafish primers		Sequence
hrpt1	forward	TTGCAGTAGCTTGTCGGTGT
hrpt1	reverse	CAGACGTTCAGTTCGGTCCA
actb2	forward	ATGGATGATGAAATTGCCGCAC
actb2	reverse	ACCATCACCAGAGTCCATCACG
ch25h	forward	CGGTGAATCCCATGTTGCTT
ch25h	reverse	AGCTCCTCCGTAAAGTCCAAAA
ch25hl1.1	forward	GTACTGCTGGCCTTCTCCAG
ch25hl1.1	reverse	GGCATAGGCATTACCACGTT
ch25hl1.2	forward	CGACTCAACCACTCAGAGACC
ch25hl1.2	reverse	AAGGTACGGCAGGACAAGAA
ch25hl2	forward	CAATGTACCTGGTGCTGGTG
ch25hl2	reverse	GCAGATGGTTGTACGTGGTG
ch25hl3	forward	TCTTCTCGGTGCCCTTCTTA
ch25hl3	reverse	AAACGCCAACCACGTATTTC
**human primers**		**Sequence**
HPRT1	forward	CAGGCGAACCTCTCGGCTTT
HPRT1	reverse	GGGTCGCCATAACGGAGCC
RNA18SN	forward	GTAACCCGTTGAACCCCATT
RNA18SN	reverse	CCATCCAATCGGTAGTAGCG
HMGCR	forward	GACGTGAACCTATGCTGGTCAG
HMGCR	reverse	GGTATCTGTTTCAGCCACTAAGG
CH25H	forward	AACGTCACACTGCTCGGGTG
CH25H	reverse	GTGGTCCTCCACGGAAAGCC
SQLE	forward	CTCCAAGTTCAGGAAAAGCCTGG
SQLE	reverse	GAGAACTGGACTCGGGTTAGCT
ABCG1	forward	GAGGGATTTGGGTCTGAACTGC
ABCG1	reverse	TCTCACCAGCCGACTGTTCTGA
**mouse primers**		**Sequence**
Hprt1	forward	GAGAGCGTTGGGCTTACCTC
Hprt1	reverse	ATCGCTAATCACGACGCTGG
Rn18s	forward	GTAACCCGTTGAACCCCATT
Rn18s	reverse	CCATCCAATCGGTAGTAGCG
Ch25h	forward	CCAGCTCCTAAGTCACGTC
Ch25h	reverse	CACGTCGAAGAAGGTCAG
**zebrafish probes**		**Assay ID**
hprt1		Dr03095135_m1
hmgcrb		Dr03128326_m1
sqlea		Dr03131215_g1
